# Neuropsychologische und Magnetresonanztomographie(MRT)-Diagnostik bei sekundär progredienter Multipler Sklerose

**DOI:** 10.1007/s00115-021-01118-9

**Published:** 2021-04-23

**Authors:** I.-K. Penner, A. Gass, H. Schreiber, M. P. Wattjes

**Affiliations:** 1 Klinik für Neurologie, Medizinische Fakultät, Heinrich-Heine-Universität Düsseldorf, Düsseldorf, Deutschland; 2 COGITO Zentrum für angewandte Neurokognition und neuropsychologische Forschung, Merowingerplatz 1, 40225 Düsseldorf, Deutschland; 3 Neurologische Klinik, Universitätsmedizin Mannheim, Mannheim, Deutschland; 4 Nervenärztliche Gemeinschaftspraxis, Neuropoint Akademie und NTD, Ulm, Deutschland; 5 Institut für diagnostische und interventionelle Neuroradiologie, Medizinische Hochschule Hannover, Hannover, Deutschland

**Keywords:** Sekundär progrediente Multiple Sklerose, Kognition, Bildgebung, Magnetresonanztomographie, Klinische Diagnostik, Secondary progressive multiple sclerosis, Cognition, Brain imaging, Magnetic resonance imaging, Clinical diagnostics

## Abstract

**Hintergrund:**

Die Multiple Sklerose ist im longitudinalen Verlauf oft ein Krankheitskontinuum mit initial schubförmig-remittierender Phase (RRMS) und späterer sekundärer Progredienz (SPMS). Die meisten bisher zugelassenen Therapien sind bei SPMS nicht ausreichend wirksam. Die frühe Erkennung der SPMS-Konversion ist daher entscheidend für die Therapiewahl. Wichtige Entscheidungshilfen können dabei die Testung kognitiver Teilleistungen und die Magnetresonanztomographie (MRT) sein.

**Ziel der Arbeit:**

Darstellung der Bedeutung kognitiver Testungen und von MRT-Untersuchungen für Prädiktion und Erfassung der SPMS-Konversion. Ausarbeitung von Strategien der Verlaufsbeobachtung und Therapiesteuerung in der Praxis, insbesondere in der ambulanten Versorgung.

**Material und Methoden:**

Übersichtsarbeit auf Basis einer unsystematischen Literaturrecherche.

**Ergebnisse:**

Standardisierte kognitive Testung kann für die frühe SPMS-Diagnose hilfreich sein und die Verlaufsbewertung erleichtern. Eine jährliche Anwendung sensitiver Screeningtests wie Symbol Digit Modalities Test (SDMT) und Brief Visual Memory Test-Revised (BVMT‑R) oder der Brief International Cognitive Assessment for MS (BICAMS)-Testbatterie ist empfehlenswert. Persistierende inflammatorische Aktivität im MRT in den ersten drei Jahren der Erkrankung sowie das Vorhandensein kortikaler Läsionen sind prädiktiv für eine SPMS-Konversion. Ein standardisiertes MRT-Monitoring auf Merkmale einer progressiven MS kann den klinisch und neurokognitiv begründeten SPMS-Verdacht stützen.

**Diskussion:**

Die interdisziplinäre Versorgung von MS-Patienten durch klinisch versierte Neurologen, unterstützt durch neuropsychologische Testung und MRT, hat einen hohen Stellenwert für die SPMS-Prädiktion und Diagnose. Letztere erlaubt eine frühe Umstellung auf geeignete Therapien, da bei SPMS andere Interventionen als für die RRMS notwendig sind. Nach erfolgter medikamentöser Umstellung erlaubt die klinische, neuropsychologische und bildgebende Vigilanz ein stringentes Monitoring auf neuroinflammatorische und -degenerative Aktivität sowie Therapiekomplikationen.

## Abstract (en)

**Background:**

Multiple sclerosis is a disease continuum with an initial relapsing remitting course (RRMS) and secondary progression (SPMS) at later stages. Most of the hitherto approved treatments do not adequately control for the phase of secondary progression. Thus, early detection of SPMS conversion is a key issue to initiate SPMS-tailored treatment. In this context, assessment of cognitive functions and magnetic resonance imaging (MRI) both play an important role in the longitudinal follow-up of MS patients.

**Objective:**

To elucidate the importance of cognitive testing and MRI for prediction and detection of SPMS conversion as well as to discuss strategies for disease monitoring and for optimizing treatment in standard clinical care, particularly in outpatient settings.

**Material and methods:**

Review article based on a nonsystematic literature review.

**Results:**

Standardized cognitive testing can support early diagnosis of SPMS and facilitate disease monitoring. Annual application of sensitive screening tests, such as the Symbol Digit Modalities Test (SDMT) and Brief Visual Memory Test-Revised (BVMT‑R) or the entire Brief International Cognitive Assessment for MS (BICAMS) test battery are recommended in this context. The MRI evidence of persistent inflammatory activity within 3 years of diagnosis as well as evidence of cortical lesions are predictive for SPMS conversion. Standardized MRI monitoring for markers of progression can substantiate clinical and neurocognitive signs of SPMS conversion.

**Conclusion:**

Multidisciplinary patient care involving careful clinical examination, neuropsychological testing and MRI monitoring is of great significance for the prediction of SPMS conversion and diagnostics. This enables early treatment adaptation, since pharmacological interventions in SPMS differ from those in RRMS. Continuous clinical, neuropsychological and MRI vigilance enable stringent monitoring of treatment outcomes with respect to neuroinflammatory and neurodegenerative activity as well as treatment-related complications.

Zentrales Risiko der Multiplen Sklerose ist die schleichende Zunahme irreversibler funktioneller Defizite im Krankheitsverlauf. Krankheitstypisch ist dabei ein initial schubförmiger Verlauf mit späterem Übergang in eine sekundäre Progredienz. Die Phase der sekundären Progredienz war bis dato therapeutisch kaum beeinflussbar. Mittlerweile hat sich die Therapielandschaft aber verändert. Die Prädiktion des Krankheitsverlaufs sowie die frühzeitige (und valide) Erkennung der SPMS-Konversion gewinnen daher immer mehr an Bedeutung. Eine wichtige Rolle für die Verlaufsbeurteilung können dabei kognitive und hirnstrukturelle Veränderungen spielen.

## Definition und Pathogenese der SPMS

Die Multiple Sklerose (MS) ist die häufigste chronisch entzündliche Erkrankung des zentralen Nervensystems (ZNS) mit einem sehr breiten Spektrum an klinischen und bildgebenden Befunden [51]. Im Verlauf präsentiert sich die klassische MS als Krankheitskontinuum, bei dem sich sehr häufig aus einer initial schubförmig-remittierenden MS (RRMS) eine sekundär progrediente Form (SPMS) entwickelt. Klinische Merkmale der SPMS sind die schubunabhängige Progression mit oder ohne aufgesetzte Schübe und das Fehlen einer vollständigen Remission. Leitlinienkonform ist sie charakterisiert durch eine schubunabhängige stetige Zunahme klinischer Symptome und neurologischer Beeinträchtigungen über mindestens sechs Monate hinweg [16]. Unterschieden wird zwischen einer aktiven und einer inaktiven SPMS anhand der Krankheitsaktivität in Form überlagerter klinischer Schübe oder entzündlicher Aktivität in der Magnetresonanztomographie (MRT) des Gehirns bzw. Rückenmarks [38, 39]. Die Relevanz einer Differenzierung zwischen RRMS und SPMS als Grundlage für mögliche Therapieentscheidungen hat mit der Zulassung von Siponimod zur Behandlung einer aktiven SPMS im Januar 2020 zugenommen.

**Figure 1:**
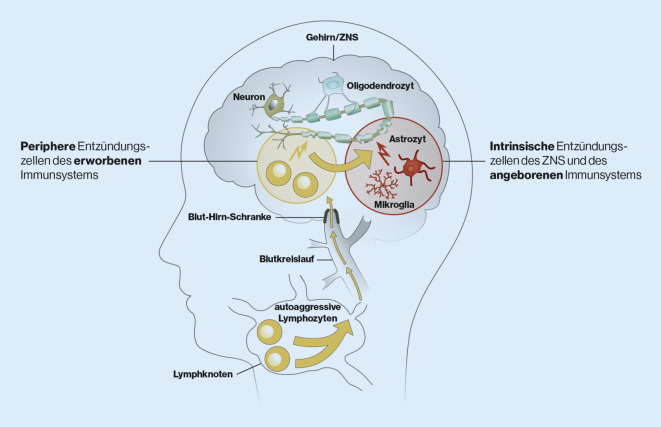

Die der schleichenden Progression zugrunde liegenden Mechanismen der SPMS sind bisher nur unvollständig verstanden (Abb. 1). Man geht aktuell davon aus, dass aufgrund eines peripher induzierten und getriebenen Entzündungsprozesses autoaggressive Lymphozyten über die beschädigte Blut-Hirn-Schranke in das ZNS eindringen. Dazu kommen Entzündungsherde innerhalb des ZNS, die unabhängig von den peripheren Entzündungsprozessen bei geschlossener Blut-Hirn-Schranke ablaufen [6, 35]. Sie scheinen an der schleichenden Krankheitsprogression wesentlich mitzuwirken [4, 15]. Diese Prozesse können bereits im frühen Krankheitsgeschehen relevant sein, werden aber oftmals nicht erkannt, da die entstehenden mikrostrukturellen Schädigungen nur mit quantitativen MRT-Methoden früh detektierbar sind [15, 23, 41, 44, 64]. Im Verlauf treten die ZNS-intrinsischen Prozesse in den Vordergrund und man kann zum Teil einen Shift von einer neuroinflammatorischen zu einer neurodegenerativen Erkrankung beobachten [15, 63].

Die Behinderungsprogression ist Folge einer unvollständigen Erholung neurologischer Funktionen und spiegelt chronische und irreversible Demyelinisierung, Axonverluste und reaktive Gliose wider [38]. Die unvollständige Erholung lässt sich auch durch erschöpfte Plastizitätsreserven des Gehirns erklären, die die Schädigung der Hirnsubstanz zu Beginn noch kompensieren. Sind diese Reserven jedoch aufgebraucht, kommt es zum Verlust sensorischer, motorischer, vegetativer und kognitiver Fähigkeiten [26].

## Klinische Diagnosestellung und Therapiesteuerung bei SPMS in der Praxis

Beim Übergang zur SPMS treten Schübe anfangs noch auf, werden dann aber meist seltener [10]. Aufgrund der überlagerten Schübe wird eine frühe schubunabhängige Progredienz (PIRA) oft nicht erkannt. So zeigte eine longitudinale Kohortenstudie über acht Jahre eine zeitliche Latenz zwischen erstem Verdacht und bestätigter SPMS-Diagnose von etwa drei Jahren [30]. Dies scheint zum einen mit der Subtilität der frühen Progression zusammenzuhängen. Zum anderen wurde teilweise trotz erkannter Progredienz wegen fehlender Alternativen die bestehende Therapie, insbesondere mit den seit längerem bei aktiver SPMS (mit aufgesetzten Schüben) zugelassenen βa-Interferonen 1b und 1a s.c., bis ins späte Krankheitsstadium fortgesetzt, in dem in der Regel die PIRA dominiert. Dies könnte erklären, warum die bisher verfügbaren klinischen Daten keinen positiven Langzeiteffekt der β‑Interferone auf die Behinderungsprogression bei SPMS belegen konnten. Therapiealternativen waren bis vor kurzem nur Mitoxantron, welches für die hochaktive MS mit Behinderungsprogression zugelassen ist, wodurch sich eine Indikation für ausgewählte Fälle mit aktiver SPMS ergibt sowie intensivierte symptomatische Behandlungen oder, bei positivem Ansprechen, regelmäßige Kortisonapplikationen (intravenös oder intrathekal). Mit einer Substanz aus dem Spektrum der S1P-Modulatoren (Siponimod) gibt es nun erstmals eine orale für die aktive SPMS zugelassene Therapie, wobei „aktiv“ durch aufgesetzte Schübe und/oder MRT-Aktivität definiert ist [20]. Dadurch erweitern sich Therapiespektrum und -anwendungen bei SPMS. Ein signifikant positiver Effekt auf die Behinderungsprogression über zwei Jahre konnte in einer SPMS-Population mit relativ hohem Ausgangs-EDSS, verstärkt bei noch vorhandener Schubaktivität, dokumentiert werden [29]. Ob sich dieser Effekt allerdings im längerfristigen Verlauf bestätigt, müssen weitere Untersuchungen zeigen. Auf jeden Fall machen diese Daten deutlich, dass eine frühzeitige und verlässliche Erkennung einer SPMS-Konversion als Basis einer Therapieentscheidung mehr denn je von Relevanz ist. Die eindeutige klinische Definition der frühen SPMS ist jedoch nach wie vor eine große Herausforderung [37]. Neben dem klinisch-neurologischen Erscheinungsbild kann die neurokognitive Testung sowie die MRT-Bildgebung hier eine wichtige Rolle, spielen [2, 22].

## Kognition bei SPMS

Kognitive Defizite wirken sich stark negativ auf die Lebensqualität und die Arbeitsfähigkeit von MS-Patienten aus [5]. Eine Verschlechterung der kognitiven Fähigkeiten ist prädiktiv für die Abnahme und den Verlust des beruflichen Status [56]. So berichten 34 % von an MS erkrankten Patienten über einen negativen Einfluss auf die Arbeitsproduktivität [33]. Nicht arbeitsfähige MS-Patienten weisen zudem eine stärkere kognitive Beeinträchtigung auf als arbeitsfähige [24]. Dabei sind der Zeitpunkt und das Ausmaß des Auftretens kognitiver Defizite höchst individuell. Sie kommen unabhängig vom Behinderungsgrad vor und können schon früh im Krankheitsverlauf in Erscheinung treten [33].

Im Stadium der sekundären Progredienz sind kognitive Defizite deutlich häufiger zu beobachten. Sie betreffen ca. 40 % der RRMS-Patienten, bei SPMS steigt der Anteil jedoch auf über 80 % [50]. Die hohe Prävalenz bei SPMS wurde in einer großen multizentrischen Studie mit einer Rate von 79,4 % bestätigt [55]. Einer weiteren Studie zufolge leiden SPMS-Patienten nicht nur etwa doppelt so häufig an kognitiven Defiziten wie RRMS-Patienten im Spätstadium, sondern auch häufiger als PPMS-Patienten [49]. Alle genannten Studien zeigen zudem, dass sich das Profil der Kognitionsstörung zwischen RRMS, SPMS und PPMS nicht qualitativ und damit nicht primär domänenspezifisch, sondern vor allem quantitativ unterscheidet. Die kognitive Verarbeitungsgeschwindigkeit ist die vulnerabelste Domäne, deren Störung sich als kognitive Verlangsamung äußert. Routinediagnostisch wurde die Kognition trotz dieser Daten bislang eher vernachlässigt und verlaufsabhängige Profile selten erhoben.

Indikatoren für kognitive Defizite in der Praxis sind vor allem kognitive Verlangsamung, Störungen des visuell-räumlichen und sprachbezogenen Kurzzeitgedächtnisses, Aufmerksamkeitsdefizite und eine exekutive Dysfunktion [47]. Faktoren wie ein höheres Alter, Konzentrationsstörungen, Fatigue, Arbeitsplatzkonflikte [14, 33, 55] sowie spezifische MRT-Veränderungen sollten für kognitive Defizite sensibilisieren (siehe „Zusammenhang von MRT und Kognition“).

Um Veränderungen in der kognitiven Leistungsfähigkeit rechtzeitig zu erkennen, empfiehlt sich eine Kognitionstestung bei Diagnosestellung und anschließend jährlich, unabhängig vom Krankheitsstadium. Dabei sollten konfundierende Einflüsse durch Fatigue, Depression und Angst berücksichtigt werden.

**Figure 2:**
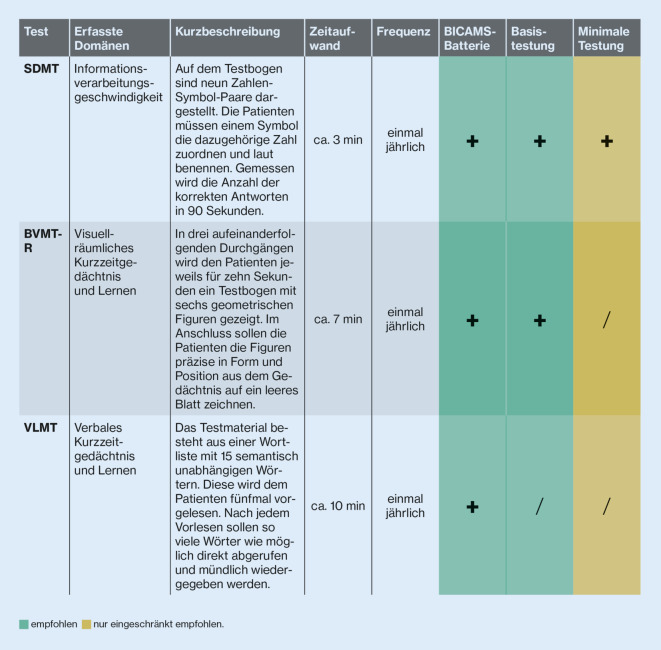

Die kognitive Leistungsfähigkeit lässt sich im niedergelassenen Bereich durch die kombinierte Anwendung von SDMT und BVMT‑R bzw. die Verwendung der BICAMS-Testbatterie mit SDMT, BVMT‑R und VLMT ausreichend gut und zuverlässig erfassen (Abb. 2). Falls dies in den Praxisalltag nicht integrierbar ist, kann der SDMT auch singulär zur Anwendung kommen. Für verlässlichere Hinweise auf die Transitionsphase RRMS/SPMS empfiehlt sich aber die Kombination, da SPMS-Patienten neben einer kognitiven Verlangsamung auch durch eine Abnahme der visuellen Gedächtnisleistung auffallen [52]. Umfangreichere Testbatterien erfordern Spezialzentren und/oder Fachpersonal wie (Neuro)Psychologen.

**Figure 3:**
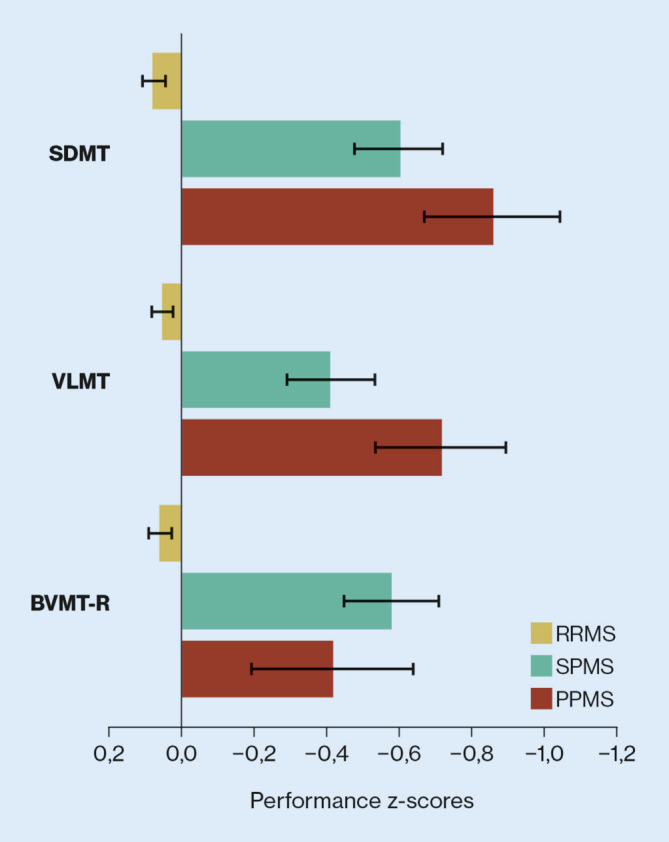

Die regelmäßige und standardisierte Testung macht eine Veränderung in kognitiven Domänen auf individueller Ebene erkennbar. Progrediente MS-Patienten schneiden in vielen kognitiven Domänen schlechter ab als Patienten mit schubförmiger MS [28]. Die größten Unterschiede zwischen RRMS- und SPMS-Patienten liegen in der kognitiven Verarbeitungsgeschwindigkeit und im visuell-räumlichen Kurzzeitgedächtnis und Lernen [28]. Das visuell-räumliche Kurzzeitgedächtnis zeigte sich hierbei auch als bester Diskriminationsfaktor zu PPMS-Patienten (Abb. 3).

## Bildgebung bei SPMS

**Figure 4:**
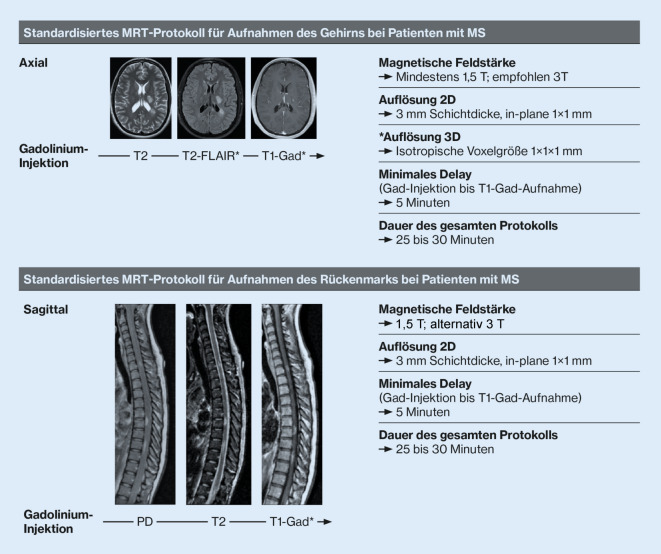

Die Standardisierung der Bildakquisition und -interpretation ist Grundvoraussetzung für das Monitoring von MS-Patienten. Protokolle für die zerebrale und spinale MRT sind international etabliert (Abb. 4; [54, 65]).

Neben frühen MRT-Markern zur Prädiktion einer Langzeitbehinderung sind weitere Marker identifiziert, die prädiktiv für eine sekundäre Progression sind, wie eine persistierende inflammatorische Aktivität in den ersten drei Jahren sowie infratentorielle, spinale und kortikale Läsionen [7, 11, 57, 60].

Die SPMS ist phänotypisch anders als die frühe RRMS. Es kommt zu einer Akzeleration der Läsionslast in der (kortikalen) grauen Substanz und im Rückenmark, zur neurodegenerativen Progression der Hirn- und Rückenmarksatrophie sowie zu mikrostrukturellen Veränderungen [18, 21, 34]. Bei SPMS-Patienten zeigt sich ein Plateau der Korrelation von Läsionslast in der weißen Substanz in Relation zu der körperlichen Behinderung [36].

Inflammatorische Läsionen, insbesondere schrankengestörte Läsionen sind seltener. Aktive (neue oder größenprogrediente) T2-Läsionen als inflammatorische Marker sind aufgrund der häufig präexistenten hohen Läsionslast schwierig zu identifizieren. MR-Subtraktionstechniken können die Sensitivität erhöhen [45]. Die Sensitivität der Detektion kortikaler Läsionen kann durch höhere magnetische Feldstärken und spezielle Pulssequenzen (z. B. „double inversion recovery“, „phase-sensitive inversion recovery“) erhöht werden [13, 59, 62]. Eine hohe Interrater-Variabilität bei fehlender Standardisierung der Befundung verhindert aber deren Implementierung in der klinischen Routine [25]. Die prognostische Relevanz und Progression der Rückenmarkspathologie im Krankheitsverlauf suggeriert die Verlaufsbeobachtung asymptomatischer Rückenmarksläsionen, insbesondere bei SPMS-Patienten [67]. Der routinemäßige Einsatz der spinalen MRT ist möglich, erfordert jedoch ein hohes Maß an Standardisierung und Expertise.

Neue inflammatorische MRT-Marker sind suggeriert worden. Leptomeningeale B‑Zell-Follikel sind insbesondere bei SPMS als Anreicherung auf kontrastverstärkten 3‑D-FLAIR-Sequenzen beschrieben worden [40, 68]. Diese Veränderungen scheinen jedoch über mehrere Jahre konstant zu sein und sind daher als Progressionsmarker ungeeignet [1, 31]. Sogenannte chronisch progrediente „Smoldering“(„slowly expanding/evolving“)-MS-Läsionen mit einem hypointensen Rand auf T2*- bzw. SWI-Sequenzen sind als charakteristisch bei SPMS und PPMS beschrieben worden [12, 19]. Aufgrund der langsamen Progredienz und der nötigen stringenten Standardisierung der Bildakquisition (v. a. Repositionierung, Auswahl der Pulssequenzen) ist eine Routineanwendung dieses Markers fraglich.

Hirn- und Rückenmarksatrophie sind insbesondere für die Prädiktion von Krankheitsprogression, vor allem kognitiver Defizite, relevant. Die Routineerhebung volumetrischer Daten erfordert neben einer stringenten Standardisierung der Bildakquisition die Einbeziehung multipler potenzieller Einflüsse (z. B. Alterungsprozess, Alkohol etc.) bei der Interpretation und nachfolgenden Therapieentscheidungen. Daher (ist) wird die Hirn- und Rückenmarksatrophie als Marker individueller Progression in der klinischen Routine derzeit nicht empfohlen [65, 66], was sich voraussichtlich auch in den nächsten Jahren nicht ändern wird. Die klinische Notwendigkeit einer Implementierung in der Vigilanz insbesondere bei SPMS-Patienten ist jedoch offensichtlich.

Komorbiditäten sind ein wichtiger Faktor für die individuelle klinische Symptomatik und das Outcome. Insbesondere eine vaskuläre Komorbidität ist bei MS-Patienten, insbesondere in späten Stadien, häufiger und prominenter als bei Gesunden [42]. Durch das sog. „zentrale Venenzeichen“ („central vein sign“) in der MRT des Gehirns kann eine vaskuläre Komorbidität von der MS-Pathologie unterschieden werden. Vaskuläre Läsionen weisen aufgrund des fehlenden perivaskulären Verteilungsmusters meist keine zentrale Vene auf [32]. Da vaskuläre Läsionen eine inflammatorische Aktivität vortäuschen können, ist die Unterscheidung zur Vermeidung nicht notwendiger Therapieentscheidungen relevant.

## Zusammenhang von MRT und Kognition

Die Relevanz der MRT wird durch die Zusammenhänge von Bildgebung und Kognition deutlich. MRT-Korrelate für kognitive Dysfunktionen sind die T2-Läsionslast, die kortikale Läsionslast und die kortikale Dicke sowie die globale und fokale Hirnatrophie [8, 9, 53]. Bestimmte Läsionslokalisationen bergen ein höheres Risiko für kognitive Defizite. Auch welche kognitive Domäne beeinträchtigt ist, wird von der Läsionslokalisation bzw. dem Verteilungsmuster mitbestimmt. Läsionen in der weißen Substanz sind maßgeblich für die kognitive Verarbeitungsgeschwindigkeit verantwortlich, Läsionen der tiefen grauen Substanz (z. B. im Hippokampus) sind häufig mit Gedächtnisdefiziten assoziiert [53].

**Figure 5:**
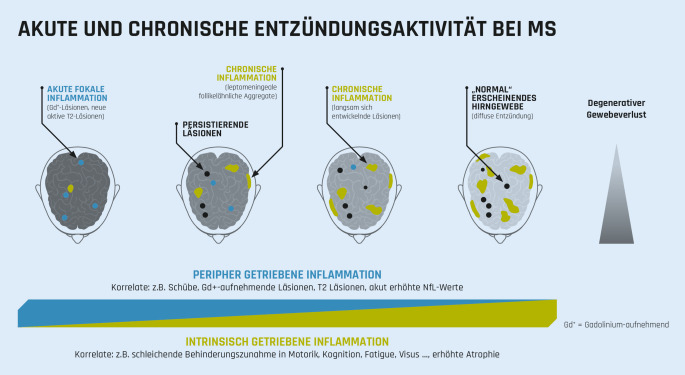

Die Interaktion der Schädigung weißer und grauer Substanz führt zu einem Netzwerkkollaps mit deutlichen kognitiven Störungen [17]. Die frühe Thalamusatrophie ist besonders bedeutend [43] und schränkt die Kommunikation zum Kortex ein. Die Mehrzahl der Läsionen liegt in den thalamokortikalen Verbindungsbahnen, was die Konnektivität subkortikal nach kortikal stört und zu einer Thalamusatrophie führen kann [61]. Das Ausmaß der Schädigungen entscheidet nicht über das Ausmaß der kognitiven Störung, da die Plastizität des Gehirns und die kognitive Reserve zunächst den Funktionsverlust kompensieren. Sind diese erschöpft kommt es zum Netzwerkkollaps mit klinischen Beeinträchtigungen (Abb. 5). Da der strukturelle Schaden aber weit früher entsteht, ist die frühzeitige therapeutische Intervention bedeutend [58].

Patienten mit SPMS mit einem geringeren Volumen des gesamten Gehirns, Thalamus und grauer Substanz haben eine schlechtere Prognose bezüglich ihrer kognitiven Leistungsfähigkeit. Auswertungen des SDMT über 24 Monaten in Abhängigkeit vom Hirnvolumen zeigen eine signifikante Verschlechterung bei Patienten mit mehr Atrophie. Ein niedriges Ausgangsvolumen von kortikaler grauer Substanz, Thalamus und gesamtem Gehirn ist signifikant assoziiert mit einem Rückgang der kognitiven Verarbeitungsgeschwindigkeit [3]. Die pathologische Belastung in der Bildgebung deutet eine höhere Vulnerabilität des Systems in der Zukunft an. Wichtig ist daher eine frühzeitige effektive Behandlung entzündlicher Prozesse, um kognitive Einschränkungen zu minimieren. Patienten mit kognitiven Funktionseinschränkungen bei MS-Diagnose weisen eine schnellere Behinderungsprogression und häufigere SPMS-Konversion auf als Patienten ohne. Die frühe kognitive Schädigung ist demnach ein Prädiktor für die Langzeitentwicklung [48].

Daten zu der 2020 für die Behandlung erwachsener Patienten mit aktiver SPMS ebenfalls zugelassenen Substanz Siponimod unterstreichen die Bedeutung einer frühzeitigen therapeutischen Intervention bei progredienter MS. So konnte das Risiko einer nach drei bzw. sechs Monaten bestätigten EDSS-Progression in der gesamten SPMS-Population statistisch signifikant um 21 % bzw. 26 %, bei aktiver SPMS mit aufgelagerten Schüben und/oder MRT-Aktivität sogar um 31 % bzw. 37 % gesenkt werden [27, 29]. Weiterhin zeigten sich komplementäre positive Ergebnisse in den MRT-Endpunkten zur entzündlichen Erkrankungsaktivität (Gd-anreichernde T1-Läsionen, aktive T2-Läsionen) und zum irreversiblen neurodegenerativen Volumenverlust (kortikale graue Substanz, Thalamus und gesamtes Gehirn). Bezüglich der Informationsverarbeitungsgeschwindigkeit gemessen mit dem SDMT konnte das Risiko einer klinisch relevanten Verschlechterung in der Verumgruppe vs. Placebo signifikant um 25 % reduziert werden [46]. Als klinisch relevant war eine Verschlechterung um 4 oder mehr Punkte definiert, was gleichbedeutend mit einer Einschränkung der Arbeitsfähigkeit ist.

## Schlussfolgerung

Die Bedeutung einer möglichst frühzeitigen Erkennung einer MS-Progression ist vor dem Hintergrund der Zulassung wirksamer neuer Therapien deutlich gewachsen. So könnten potenziell Plastizitätsreserven langfristig erhalten und funktionelle Defizite minimiert werden. Dies gilt sowohl für die schubförmig progredienten Verläufe als auch für die Konversion zur SPMS, bei der schubunabhängige Progression (PIRA) vorherrscht.

In der klinischen Praxis ist dafür im ersten Schritt eine zeitlich optimierte Progressionsdiagnostik notwendig. Dies kann erreicht werden, wenn neben der Symptomanamnese und den motorischen Parametern, wie Gehstrecke und EDSS, auch kognitive Funktionen systematisch untersucht werden. Weiterhin ermöglicht eine standardisierte MRT-Untersuchung nach neuesten internationalen Konsensusrichtlinien die bestmögliche Verlaufsbeurteilung und eröffnet damit Möglichkeiten zu einem besseren Verständnis des individuellen Beeinträchtigungsprofils. Für die kognitive Testung bieten sich jeweils jährliche Erhebungen mit SDMT und BVMT‑R oder der Gesamt-BICAMS-Testbatterie an. Hinsichtlich der Bildgebung bleibt auch bei länger zurückliegender MS-Diagnose eine jährliche Erfassung der entzündlichen Aktivität im ZNS mittels Gd-anreichernder T1-Läsionen sowie aktiver T2-Läsionen Minimalstandard. Neben der individuellen Patientenperspektive können die vorgenannten Parameter im Einzelfall zur Beurteilung des Erkrankungsverlaufes im Hinblick auf den Therapieerfolg eingesetzt werden.

## Fazit für die Praxis

- Frühes Erkennen der MS-Progression allgemein und der SPMS-Konversion im Speziellen und gezieltes therapeutisches Eingreifen sind wichtig, um funktionellen Defiziten langfristig vorzubeugen.
- Eine einmal jährliche kognitive Testung mittels SDMT und BVMT‑R oder BICAMS liefert relevante Informationen zur Diagnose und Verlaufsbeurteilung.
- Eine einmal jährliche MRT-Darstellung Gd-anreichernder T1-Läsionen sowie aktiver T2-Läsionen ist auch bei SPMS relevant. Neue MRT-Marker sind noch nicht in der klinischen Routine verfügbar.
- Sensibilisierung und Awareness für das Zusammenspiel von klinischen, neuropsychologischen und MRT-Parametern ermöglichen zukünftig ein besseres Monitoring der Patienten im Hinblick auf neuroinflammatorische und neurodegenerative Aktivität sowie potenzielle Therapiekomplikationen.
